# Supplementary material for: Viral subversion of selective autophagy is critical for biogenesis of virus replication organelles
Source: Nat Commun. 2023 May 10;14:2698. doi: 10.1038/s41467-023-38377-w (PMC10171163; doi:10.1038/s41467-023-38377-w)

Viral subversion of selective autophagy is critical for biogenesis of virus replication organelles

Supplementary information:

- Supplementary figures (1-6)
- Table S1: RNAi accession codes
- Unprocessed blots (supplementary figure 6)

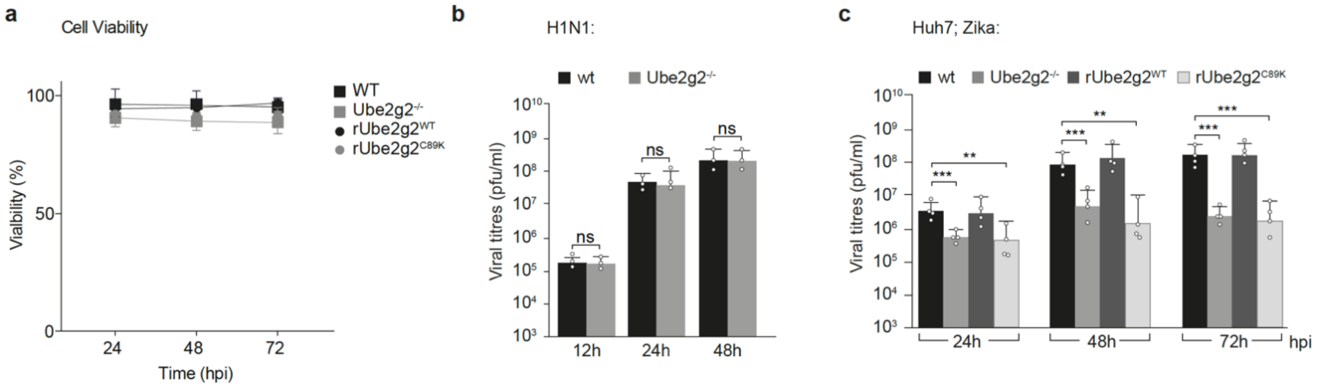

Supplementary figure 1: Ube2g2 is necessary for (+) RNA virus replication

a. Cytotoxicity of WT, Ube2g2$^{-/-}$, rUbe2g2^{WT} and rUbe2g2^{C89K} cells was measured using MTT assay as per the manufacturer's protocol. Error bars represent mean ± SD from three independent experiments. **b**. Wild-type and Ube2g2$^{-/-}$ cells were infected with H1N1pdm stain at MOI=0.1. Viral production was measured by plaque assay. Data are presented as mean ± SD, n=3 biological replicates. Statistical significance derived using ANOVA followed by one-sided Dunnett's test. **c**. Wild-type, Ube2g2$^{-/-}$, rUbe2g2^{WT} and rUbe2g2^{C89K} cells were challenged with Zika virus at MOI 5. Infectious virus production was measured using plaque assay. Data is presented as mean ± SD, n=4 biological replicates. $^{**}P < 0.01$; $^{***}P < 0.001$ (compared with wild-type by ANOVA followed by one-sided Dunnett's test).

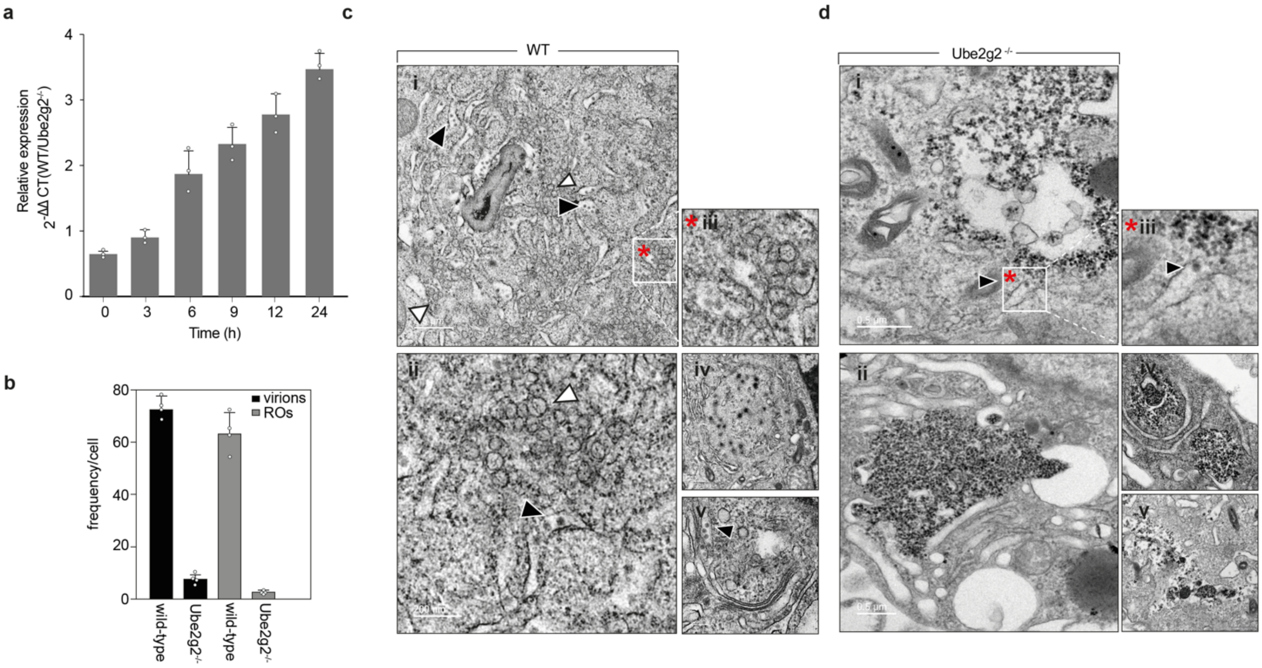

Supplementary figure 2: Ube2g2 is necessary for virus replication

a. Wild-type and Ube2g2$^{-/-}$ cells were infected with Zika at MOI=5. The total RNA from cell lysates was extracted at the indicated time points, and viral production was measured by qPCR. Internal GAPDH control was used to calculate ΔCp values. Relative expression of intracellular viral gene was presented as $2^{\Delta\Delta Cp}$ (Wild-type vs Ube2g2$^{-/-}$). Data is presented as mean ± SD, n=3 biological replicates. **b**. Quantitation of the frequency of intact virions per cell in wild-type and Ube2g2$^{-/-}$ cells. Error bars represent mean ± SD, n=4 biological replicates. **c, d.** TEM sections of Zika-infected wild-type and Ube2g2$^{-/-}$ cells. The filled black arrows indicate intact progeny virions, white arrows indicate replication organelles and convoluted membranes. Panel c(iii) and d(iii) indicates magnified versions of insets in panel c(i) and d(i) respectively.

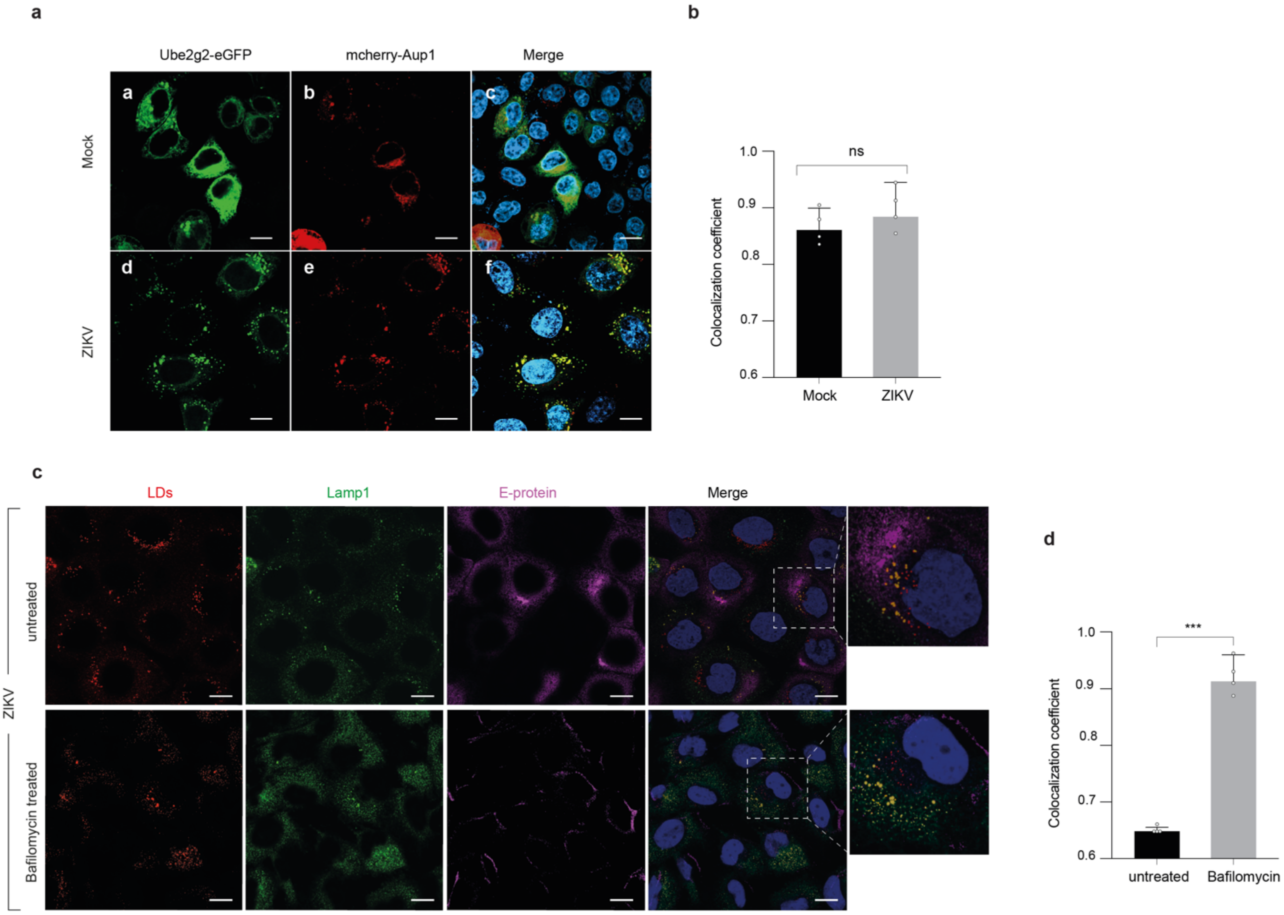

Supplementary figure 3: Aup1-Ube2g2 complex drive lipophagy during infection

a, b. Wild-type cells stably expressing Ube2g2-eGFP and mcherry-Aup1 were seeded on 24-well glass coverslips and either mock infected or infected with ZIKV at MOI 5 and fixed with 4% PFA at 24 hours. Aup1 and Ube2g2 were visualized using Carl Zeiss LSM 780/980 confocal microscope; images were analysed using ZEISS ZEN and colocalization was determined using Pearson's correlation coefficient *(Scale bars=10μm)*. Error bars represent mean ± SD, n=4 biological replicates; statistical significance derived using ANOVA followed by one-sided Dunnett's test. **c, d**. Wild-type cells treated with DMSO only or with Bafilomycin A1 (100 nM in DMSO) were infected with ZIKV (MOI 5, 24 h), fixed with 4% PFA and visualised with Nile Red, anti-Lamp1 and anti-E antibodies. Colocalization of LDs with Lamp1 was determined using Pearson's correlation coefficient *(Scale bars=10μm)*. Data is presented as mean ± SD, n=4 biological replicates. **$P < 0.01$; ***$P < 0.001$ (compared with wild-type by ANOVA followed by one-sided Dunnett's test).

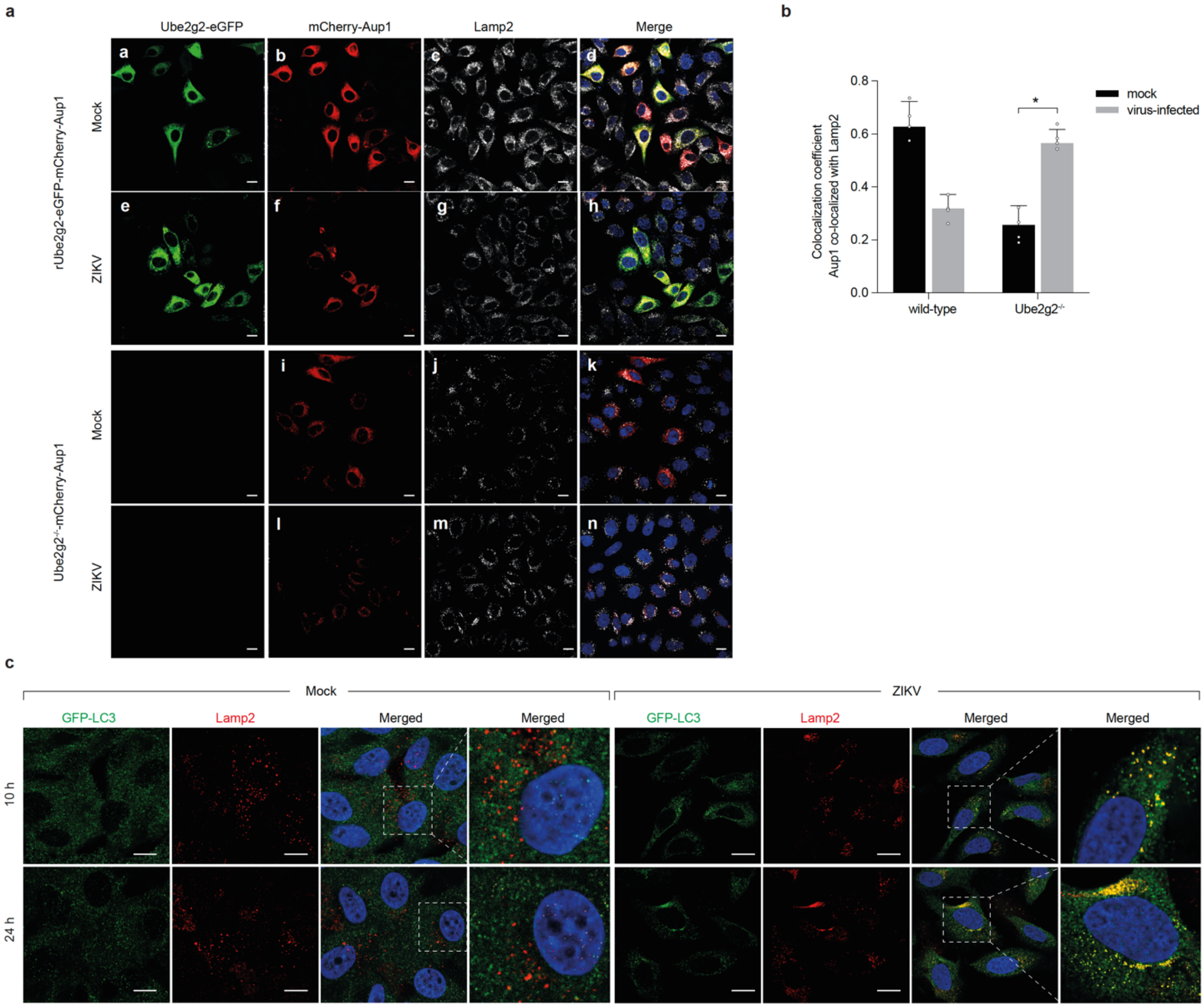

Supplementary figure 4: Ube2g2-deficiency leads to increase in autolysosomes

a. Ube2g2$^{-/-}$ cells stably expressing Ube2g2-eGFP and mcherry-Aup1 or empty lentivirus and mcherry-Aup1 were seeded on 24-well glass coverslips and either mock infected or infected with ZIKV at MOI 5 and fixed with 4% PFA at 24 hours. Aup1, Ube2g2 and Lamp2 were visualized using Carl Zeiss LSM 780/980 confocal microscope *(Scale bars=10μm)*. **b**. Images were analysed using ZEISS ZEN and colocalization was determined using Pearson's correlation coefficient. Error bars represent mean ± SD, n=4 biological replicates; statistical significance derived using ANOVA followed by one-sided Dunnett's test. **c.** Wild-type cells stably expressing GFP-LC3 were either mock-infected or ZIKV-infected for 10h or 24h. GFP and Lamp2 were visualised using Carl Zeiss LSM 780/980 confocal microscope. Insets bounded by dashed boxes are magnified for visualizing merged panels *(Scale bars=10μm)*.

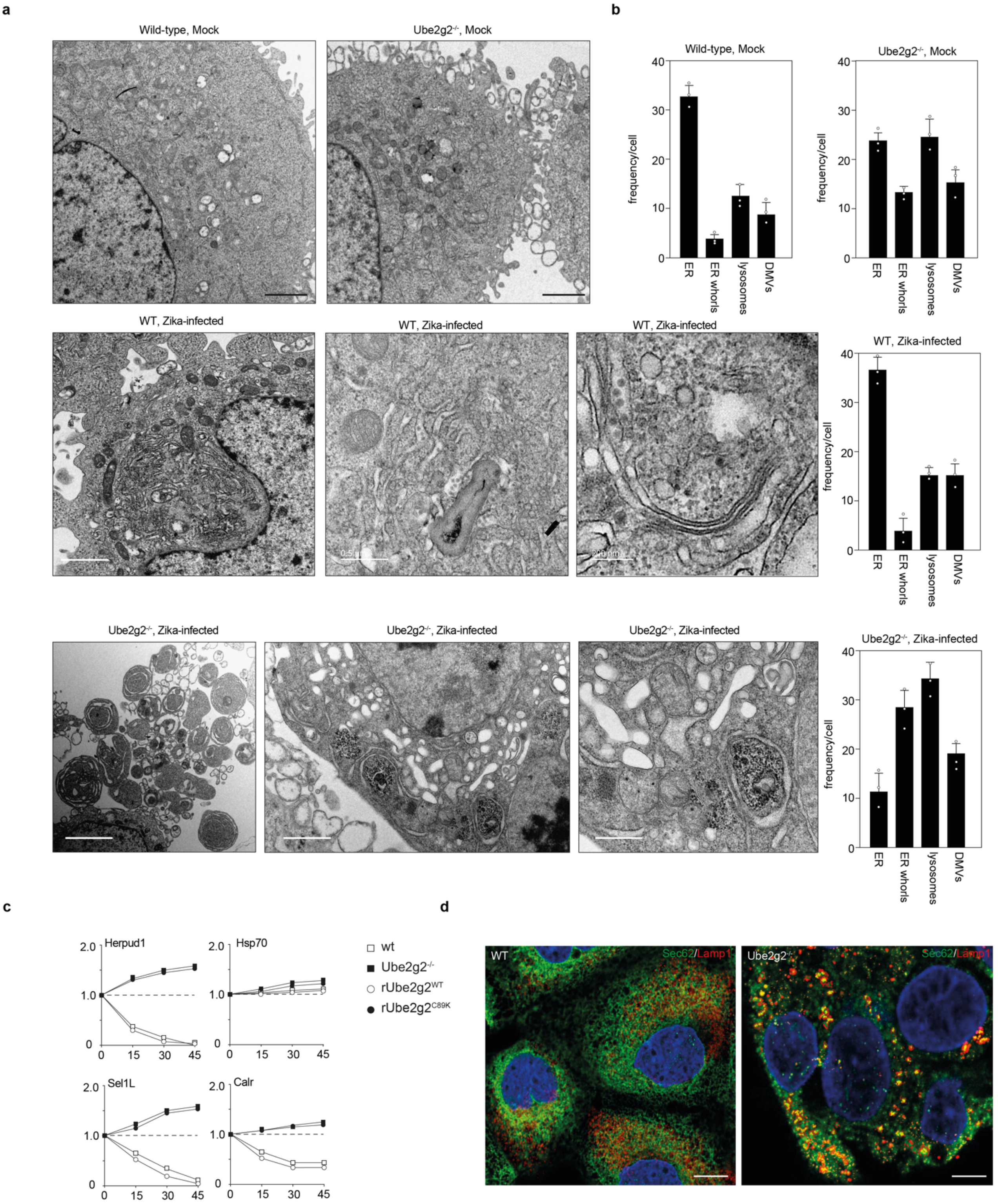

Supplementary figure 5: Aberrant intracellular organelle distribution in Ube2g2$^{-/-}$ cells

a. TEM sections of mock and Zika-infected wild-type and Ube2g2$^{-/-}$ cells. The frequency of indicated organelles was determined per cell for >20 images. **b**. Quantitation of the frequency of indicated organelles per cell in wild-type and Ube2g2$^{-/-}$ cells. Error bars represent mean ± SD, n=3 biological replicates. **c.** Turnover of newly synthesised ER proteins indicated was

quantitated using pulse chase in [^{35}S]cysteine/methionine labelled cells. **d.** Sec62 and Lamp1 were visualised in wild-type and Ube2g2$^{-/-}$ cells for measuring ER-phagy, using Carl Zeiss LSM 780/980 confocal microscope *(Scale bars=10μm)*.

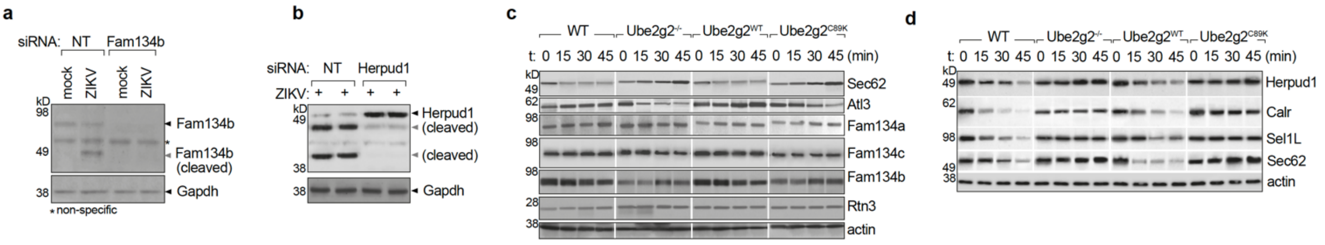

Supplementary figure 6: Aberrant degradation of ER genes in Ube2g2-deficient cells

a, b. Immunoblots were performed in lysates from mock and ZIKV-infected cells expressing non-targeting siRNA as control or against Fam134b or Herpud1. Gapdh was measured as loading control. **c, d**. Newly synthesised ER proteins as indicated were metabolically labelled with [^{35}S]cysteine/methionine in ZIKV-infected cells (b) or NS2b-NS3-expressing cells (c) and chased for indicated timepoints; turnover of indicated proteins was detected by autoradiography.

Table S1: RNAi Accession Codes

siRNA constructs

Gene	Accession Number	siRNA (SMARTpool)
Human Sec62	NM_003262	L-010218-01-0020
Human Chmp4	NM_176812	L-018075-01-0020
Human Ube2g2	NM_182688	L-009095-00-0020
Human Ube2g2	NM_003343	L-009095-00-0020
Non-targeting		A-001206

Unprocessed blots

Figure S6

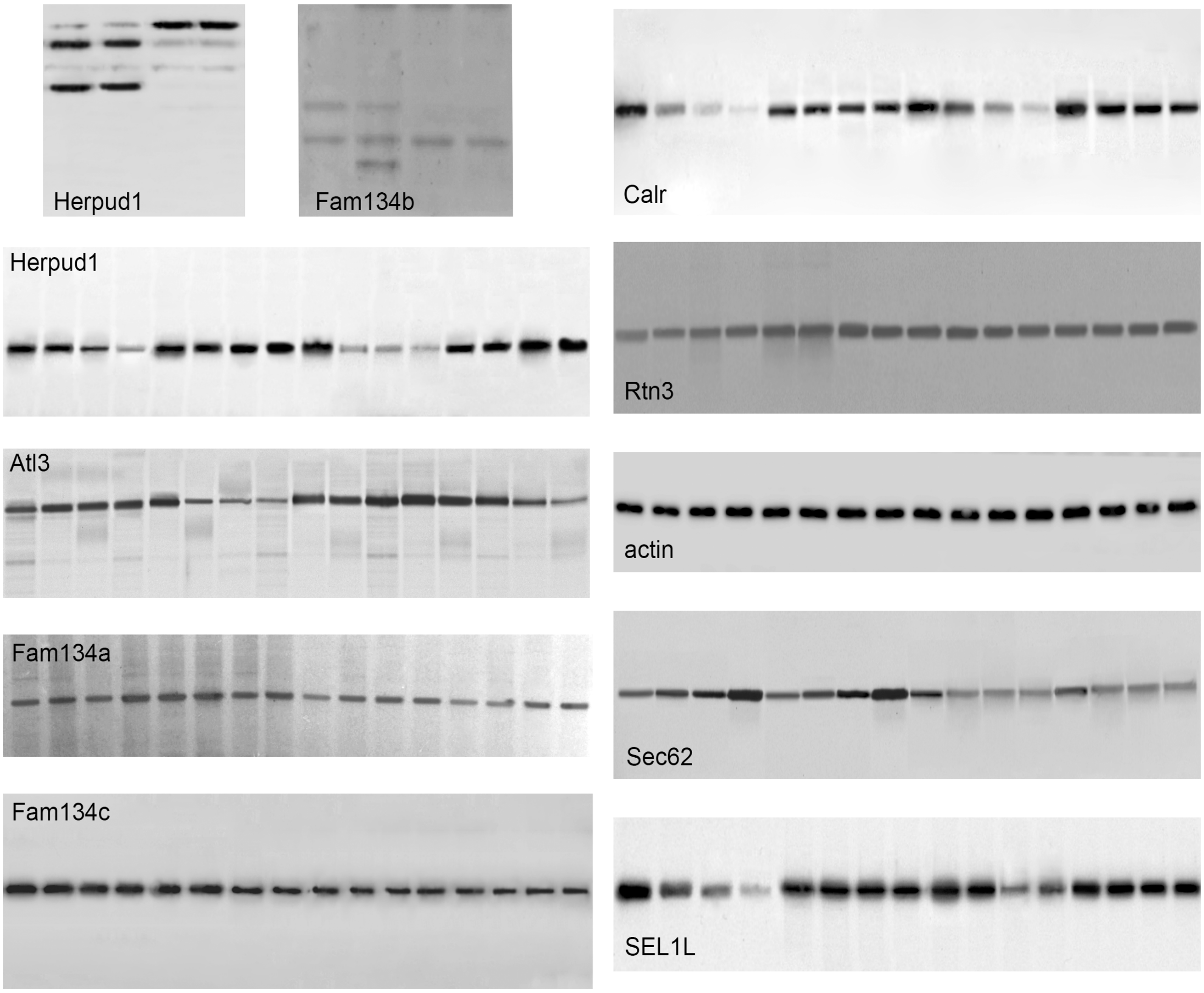

Supplement: Supplementary file 1 — Supplementary Information [file 41467_2023_38377_MOESM1_ESM.pdf]
