# Supplementary material for: Viral subversion of selective autophagy is critical for biogenesis of virus replication organelles
Source: Nat Commun. 2023 May 10;14:2698. doi: 10.1038/s41467-023-38377-w (PMC10171163; doi:10.1038/s41467-023-38377-w)
Supplement: Supplementary file 3 — Reporting Summary [file 41467_2023_38377_MOESM3_ESM.pdf]

nature portfolio

Corresponding author(s): Sumana Sanyal

Last updated by author(s): Apr 11, 2023

Reporting Summary

Nature Portfolio wishes to improve the reproducibility of the work that we publish. This form provides structure for consistency and transparency in reporting. For further information on Nature Portfolio policies, see our Editorial Policies and the Editorial Policy Checklist.

Statistics

For all statistical analyses, confirm that the following items are present in the figure legend, table legend, main text, or Methods section.

n/a	Confirmed	
☐	☒	The exact sample size (n) for each experimental group/condition, given as a discrete number and unit of measurement
☐	☒	A statement on whether measurements were taken from distinct samples or whether the same sample was measured repeatedly
☐	☒	The statistical test(s) used AND whether they are one- or two-sided<br>*Only common tests should be described solely by name; describe more complex techniques in the Methods section.*
☒	☐	A description of all covariates tested
☐	☒	A description of any assumptions or corrections, such as tests of normality and adjustment for multiple comparisons
☐	☒	A full description of the statistical parameters including central tendency (e.g. means) or other basic estimates (e.g. regression coefficient) AND variation (e.g. standard deviation) or associated estimates of uncertainty (e.g. confidence intervals)
☐	☒	For null hypothesis testing, the test statistic (e.g. F, t, r) with confidence intervals, effect sizes, degrees of freedom and P value noted<br>*Give P values as exact values whenever suitable.*
☒	☐	For Bayesian analysis, information on the choice of priors and Markov chain Monte Carlo settings
☒	☐	For hierarchical and complex designs, identification of the appropriate level for tests and full reporting of outcomes
☒	☐	Estimates of effect sizes (e.g. Cohen's d, Pearson's r), indicating how they were calculated

Our web collection on statistics for biologists contains articles on many of the points above.

Software and code

Policy information about availability of computer code

Data collection	qRT-PCR: LightCycler 48 0 software l.5.0SP3<br>imaging: Confocal microscope ZEISS ZEN Microscope<br>Attune NxT acoustic focusing cytometer (Therma Fisher, MA, USA)
Data analysis	Imaging: Zen black software version Zen 2.6 (blue edition)<br>qRT-PCR analyses: Microsoft Excel version 16.36.<br>Densitometry: ImageJ and Adobe Photoshop version 21.1.2<br>FlowJo (OSX64-10.5.3)<br>Statistical analyses: GraphPad Prism version 9.3.1 and Microsoft Excel 2016/version 16.36

For manuscripts utilizing custom algorithms or software that are central to the research but not yet described in published literature, software must be made available to editors and reviewers. We strongly encourage code deposition in a community repository (e.g. GitHub). See the Nature Portfolio guidelines for submitting code & software for further information.

Data

Policy information about availability of data

All manuscripts must include a data availability statement. This statement should provide the following information, where applicable:

- Accession codes, unique identifiers, or web links for publicly available datasets
- A description of any restrictions on data availability
- For clinical datasets or third party data, please ensure that the statement adheres to our policy

A reporting summary for this article is available as Supplementary Information file. Protein sequences used in this study were extracted from spectra that were searched against the human protein sequences in the Swiss-Prot database (database release version of May 2022), containing 20,621 sequences (www.uniprot.org) (Human; including isoforms and unreviewed sequences). Accession codes of RNAi experiments are NM_003262 for Sec62, NM_176812 for Chmp4b and NM_182688 for Ube2g2 with their corresponding catalogue number provided in the supplementary information. Data and immunoblots presented in the manuscript are provided as a Source Data file. Data generated or analysed during the current study are available through Figshare.

Human research participants

Policy information about studies involving human research participants and Sex and Gender in Research.

Reporting on sex and gender	Not applicable
Population characteristics	Not applicable
Recruitment	Not applicable
Ethics oversight	Not applicable

Note that full information on the approval of the study protocol must also be provided in the manuscript.

Field-specific reporting

Please select the one below that is the best fit for your research. If you are not sure, read the appropriate sections before making your selection.

☒ Life sciences ☐ Behavioural & social sciences ☐ Ecological, evolutionary & environmental sciences

For a reference copy of the document with all sections, see nature.com/documents/nr-reporting-summary-flat.pdf

Life sciences study design

All studies must disclose on these points even when the disclosure is negative.

Sample size	Samples sizes were selected keeping in mind the variability between independent sources of cells. All experiments were performed in mammalian cell cultures, which are population-based, with data points generated from experiments performed from cells generated from independent clones and performed independent of each other. Sample sizes were determined based on the numbers required to achieve statistical significance using indicated statistics, but with a minimum of 3 independently performed experiments to ensure data reproducibility.
Data exclusions	No data was excluded.
Replication	For all experiments data were generated with three or four independent biological replicates along with atleast 2 technical replicates to calculate the mean and standard deviation. All attempts at replication were successful.
Randomization	Cells from different clones were independently replicated on atleast 3 separate days, with wells in microtiter plates randomised for treatment conditions, and measurements taken on ~1 x E6 cells from each well.
Blinding	Blinding was not performed since cells were exposed to infectious pathogens.

Reporting for specific materials, systems and methods

We require information from authors about some types of materials, experimental systems and methods used in many studies. Here, indicate whether each material, system or method listed is relevant to your study. If you are not sure if a list item applies to your research, read the appropriate section before selecting a response.

Materials & experimental systems

n/a	Involved in the study
☐	☒ Antibodies
☐	☒ Eukaryotic cell lines
☒	☐ Palaeontology and archaeology
☒	☐ Animals and other organisms
☒	☐ Clinical data
☒	☐ Dual use research of concern

Methods

n/a	Involved in the study
☒	☐ ChIP-seq
☒	☐ Flow cytometry
☒	☐ MRI-based neuroimaging

Antibodies

Antibodies used

Rabbit α-Aup1 Ab101984 (Abcam, UK; 1:1000); Rabbit α-Ube2g2 Ab174296 (Abcam, UK; 1:1000); Rabbit α-Perilipin Ab172907 (Abcam, UK; 1:1000); Rabbit α-Hrd1 LS-C668919 (LSBio, WA, USA; 1:1000); Rabbit α-Gp78 ab101284 (Abcam, UK; 1:1000); Rabbit α-Herpud1 26730 (Cell Signalling Technology, MA, USA; 1:500); Rabbit α-LC3b 2775S (Cell Signalling Technology, MA, USA; 1:1000); α-Rabbit LAMP1 (D2D11) 9091S (Cell Signalling Technology, MA, USA; 1:1000); Mouse recombinant α-(linkage specific K63)-ubiquitin Ab179434 (Abcam, UK; 1:1000); Mouse recombinant α-(linkage specific-K48) ubiquitin Ab140601 (Abcam, UK; 1:1000); Mouse α-Ubiquitin Ab7780 (Abcam, UK; 1:1000); Rabbit polyclonal α-FAM134C ab202125 (Abcam, UK; 1:1000); α-Reticulon-3 (A302-860A, Cambridge Bioscience Ltd; 1:1000); α-FAM134B (61011), (Cat no 61011S, Cell Signaling Technology; 1:1000); HSP70 (10995-1-AP, Proteintech Europe Ltd; 1:1000); Mouse α-4G2 In-house produced (1:5000); Mouse α-GAPDH; Goat α -mouse Alexafluor®488 Ab150117 (Abcam, UK; 1:10000); Goat α-rabbit Alexafluor®488 Ab150077 (Abcam, UK; 1:10000); Goat α-mouse Alexafluor®647 Ab150119 (Abcam, UK; 1:10000); Goat α-rabbit HRP 7074 (Cell Signalling Technology, MA, USA; 1:10000); Goat α-mouse HRP Ab97040 (Abcam, UK; 1:10000); Goat α-Rabbit Texas Red® Ab6719 (Abcam, UK; 1:10000); Rabbit α-prM GTX133305 (GeneTex, USA; 1:5000); Rabbit α-NS3 GTX133309 (GeneTex, USA; 1:1000); Rabbit α-NS4A GTX133704 (GeneTex, USA; 1:5000); Rabbit α-NS4B GTX133311 (GeneTex, USA; 1:1000); Rabbit α-NS1 GTX133307 (GeneTex, USA; 1:1000); Rabbit α-NS5 GTX638132 (GeneTex, USA; 1:1000); Rabbit α-NS2B GTX GTX133308 (GeneTex, USA; 1:500); Rabbit α-Capsid GTX GTX133317 (GeneTex, USA; 1:1000)

Validation

-AUP1 detects endogenous human Aup1; Immunogen is synthetic peptide corresponding to a region between residue 426 and 476 of Human Ancient ubiquitous protein 1 (NP_853553.1). Predicted to work with: Chimpanzee, Rhesus monkey, Gorilla, Orangutan. Suitable for: WB, IP, IHC-P
-UBE2G2 Rabbit monoclonal [EPR9248(2)] to Ube2G2; Immunogen: Synthetic peptide within Human Ube2G2 aa 1-100; Reacts with: Mouse, Rat, Human; Suitable for: ICC/IF, WB, IP
-Perilipin: Rabbit monoclonal [EPR3753(2)] to Perilipin-1; Suitable for: WB, ICC/IF; Species reactivity
Reacts with: Human; Immunogen: Synthetic peptide within Human Perilipin-1 aa 450 to the C-terminus (Cysteine residue).
-LC3 detects endogenous levels of total LC3B protein. Cross-reactivity may occur with other LC3 isoforms. Stronger reactivity is observed with the type II form of LC3B. Species Reactivity: Human, Mouse, Rat. Species predicted to react based on 100% sequence homology: Monkey, Bovine, Pig. Applications: WB, IHC, IF, Flow Cytometry,
-LAMP2 reacts with Mouse, Human, Monkey, Chinese hamster. Predicted to work with: Dog. Positive control WB: MEF1 whole cell lysate and mouse lung, human liver, and human liver (membrane fraction) tissue lysates. Suitable for: WB, ICC/IF, Flow Cyt, IHC-P.
-LAMP1 recognizes endogenous levels of total LAMP1 protein. Species Reactivity: Human, Monkey. Applications WB, IP, IHC, IF, Flow Cytometry.
- HRD1: Recognizes endogenous levels of Hrd1 protein; reacts with Human, Mouse, Rat; suitable for Western blot (1:500 - 1:2000) Immunoprecipitation (1:50 - 1:100)
-GP78: Reacts with: Human; predicted to work with: Chimpanzee, Rhesus monkey, Orangutan; Suitable for: WB, IP; Immunogen: synthetic peptide corresponding to Human gp78 aa 500-550
- HERPUD1 recognizes endogenous levels of total HERPUD1 protein. This antibody does not cross-react with HERPUD2 protein; Species Reactivity: Human, Mouse, Rat, Monkey
- RTN3: Rabbit polyclonal, detects endogenous protein levels; suitable for WB, IHC, IP; species reactivity: human
- Ubiquitin (linkage-specific K63) antibody; Suitable for: Flow Cyt (Intra), WB, IHC-P; Reacts with: Mouse, Rat, Human; Immunogen: synthetic peptide within Human Ubiquitin aa 50-150 (linkage-specific K63) (Cysteine residue).
- Ubiquitin (K48): only recognizes polyubiquitin chains formed by Lys-48 (K48) residue linkage. This antibody can detect the target in mouse and rat cell lines and induced tissues; Suitable for: Flow Cyt (Intra), ICC/IF, IHC-P, WB; Reacts with: Mouse, Rat, Human
- Ubiquitin: Rabbit polyclonal to Ubiquitin; Suitable for: IHC-P, ICC/IF, IHC-FrFl; Reacts with: Rat, Human; predicted to work with: Mouse, Horse, Cow, Monkey, African green monkey
- FAM134C: Rabbit polyclonal to FAM134C - C-terminal; Suitable for: WB, IP; Reacts with: Human, predicted to work with: Mouse, Sheep, Horse, Cow, Dog, Pig, Chimpanzee, Rhesus monkey, Orangutan; validated in HeLa, 293T, and Jurkat whole cell lysate.
-FAM134B: Antibody recognizes endogenous levels of total FAM134B protein, species Reactivity: Human; validated in HeLa and 293T cell lysates
- Flavivirus glycoprotein: validated in previous publications (Zhang et al, Cell Host Microbe 2018, Li et al, Nat commun, 2020)
- anti-mouse: antibody reacts specifically with mouse IgG and with light chains common to other mouse immunoglobulins. No antibody was detected against non-immunoglobulin serum proteins. Reduced cross-reactivity to bovine, chicken, horse, human, pig, rabbit and rat IgG was detected. This antibody may cross react with IgG from other species.
- GAPDH: This GAPDH antibody can be used as a loading control antibody. There is no cross-reaction with GAPDH from yeast. Preliminary data indicates that the GAPDH antibody- loading control ab8245 recognizes the monomer (36 kDa) and also the dimer forms of GAPDH, but not the tetrameric form of the protein; Suitable for: WB, ICC/IF; Reacts with: Mouse, Rat, Human
Predicted to work with: Horse, Chicken, Guinea pig, Hamster, Cat, Dog, Pig, Xenopus laevis, Fish, Monkey, Zebrafish, Baboon, Xenopus tropicalis; Does not react with: Goat, Cow, Saccharomyces cerevisiae
- prM: Rabbit polyclonal; suitable for WB, ICC/IF, IP; validated in Zika virus(PRVABC59)-infected Vero , Zika virus prM-transfected C6/36
- NS3: Rabbit polyclonal; suitable for WB, ICC/IF, IHC-P; validated in Zika virus(PRVABC59)-infected Vero , BHK21, C6/36

- NS4A: Rabbit polyclonal; suitable for WB, ICC/IF, IP; validated in Zika virus(PRVABC59)-infected Vero , BHK21, C6/36; transfected 293T cells
- NS4B: Rabbit polyclonal; suitable for WB, ICC/IF, IHC-Fr, FACS, IP; validated in Zika virus(PRVABC59)-infected Vero , BHK21, C6/36; transfected 293T cells
- NS1: Rabbit polyclonal; suitable for WB, ICC/IF, ELISA, Sandwich ELISA; validated in Zika virus(PRVABC59)-infected Vero , BHK21, C6/36; transfected 293T cells
- NS5: Rabbit monoclonal; suitable for WB; validated in Zika virus(PRVABC59)-infected Vero , BHK21, C6/36; transfected 293T cells
-NS2B: Rabbit polyclonal; suitable for WB, ICC/IF, IHC-P, IHC-Fr, IHC-P; validated in Zika virus(PRVABC59)-infected Vero , BHK21, C6/36; transfected 293T cells
- Capsid: Rabbit polyclonal; suitable for WB, ICC/IF, IHC-P (cell pellet); validated in Zika virus(PRVABC59)-infected Vero , BHK21, C6/36; transfected 293T cells

Eukaryotic cell lines

Policy information about cell lines and Sex and Gender in Research

Cell line source(s)	Hela cells, Cat no ATCC®, CCL-2™, Huh7 cells, Merck Cat no 01042712; HeLa Ube2g2-/- (this study), HeLa rUbe2g2(WT) (this study); HeLa rUbe2g2-C89K (this study); HeLa rUbe2g2-C-FLAG (this study); HeLa rUbe2g2-C89K-C-FLAG (this study); HeLa ZIKV-PrME (Li et al, Nat Commun; 2020, generated by Mingyuan Li, HKU-Pasteur Research Pole, HKU); HeLa-Difluo™-hLC3 Human cervical cancer cell line stably expressing the RFP::GFP::LC3 fusion protein; HeLa-Difluo™-hLC3 Ube2g2-/- HeLa-Difluo™-hLC3 Ube2g2 knockout cell line (this study); HeLa-Ube2g2-eGFP-Aup1-mCherry HeLa stably expressing Ube2g2 flanked with eGFP (this study); HeLa-Ube2g2-eGFP-mCherry-Aup1 HeLa stably expressing Ube2g2 flanked with eGFP and Aup1-mCherry (this study); Vero Monkey kidney epithelial cell line (ATCC; CCL-81); C6/36 Mosquito Aedes albopictus (ATCC; CRL-1660); HEK 293T (ATCC; CRL-3216).
Authentication	All cell lines were purchased from ATCC, which were characterized and authenticated by short tandem repeat (STR) DNA profiles.
Mycoplasma contamination	all cell lines tested and confirmed to be negative for mycoplasma
Commonly misidentified lines (See ICLAC register)	No commonly misidentified cell lines (reported by ICLAC) have been used in this study.
